# Structural Information on Supramolecular Copper(II) β-Diketonate Complexes from Atomic Force Microscopy and Analytical Ultracentrifugation

**DOI:** 10.1021/acsomega.3c07493

**Published:** 2024-01-04

**Authors:** Jonathan S. Casey, Ashley R. Walker, Xianglin Zhai, Jayne C. Garno, Paul S. Russo, Andrew W. Maverick

**Affiliations:** ¶ Department of Chemistry, Louisiana State University, Baton Rouge, Louisiana 70803, United States; ⊥ Department of Chemistry and Macromolecular Studies Group, Louisiana State University, Baton Rouge, Louisiana 70803, United States

## Abstract

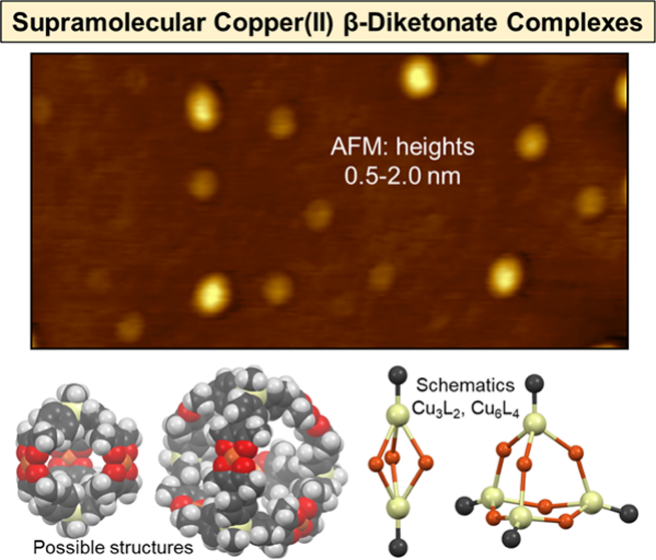

Supramolecular Cu(II) complexes were prepared from two trifunctional β-diketone ligands. The ligands (CH_3_Si(phacH)_3_ and CH_3_Si(phprH)_3_, represented by LH_3_) contain three aryl-β-diketone moieties joined by an organosilicon group. The complexes have the empirical formula Cu_3_L_2_, as expected for combinations of Cu^2+^ and L^3–^. Several metal–organic polyhedra (MOPs) [Cu_3_L_2_]*_n_* are possible (*n* = 1–10); a dodecahedron (Cu_30_L_20_; *n* = 10; estimated diameter of ca. 5 nm) should be the most stable because its internal bond angles would come closest to ideal values. Atomic force microscopy (AFM), performed on samples deposited from solution onto mica substrates, revealed a distribution of sample heights in the 0.5–3.0 nm range. The most commonly observed heights were 0.5–1.5 nm, corresponding to the smallest possible molecules (Cu_3_L_2_, i.e., *n* = 1). Some molecular cubes (Cu_12_L_8_; ca. 2.5 nm) or larger molecules or aggregates may be present as well. Equilibrium analytical ultracentrifugation (AUC) was also used to probe the compounds. A previously reported reference compound, the molecular square Cu_4_(*m*-pbhx)_4_ (*M* = 2241 g mol^–1^), behaved well in AUC experiments in four nonpolar organic solvents. AUC data for the new tris(β-diketonate) MOPs [Cu_3_L_2_]*_n_* in toluene and fluorobenzene did not agree well with the theoretical results for a single solute. The data were fit well by a two-solute model, but these results were not consistent in the two solvents used, and some run-to-run variability was noted even in the same solvent. Also, the calculated molecular weights differed significantly from those expected for [Cu_3_L_2_]*_n_* ([Cu_3_(CH_3_Si(phac)_3_)_2_]*_n_*, multiples of 1322 g mol^–1^; or [Cu_3_(CH_3_Si(phpr)_3_)_2_]*_n_*, multiples of 1490 g mol^–1^).

## Introduction

Much progress has been made in the preparation and study of metal–organic materials (MOMs) over the last two decades.^1,2^ These include metal–organic frameworks (MOFs, with < extended structures) and molecular species (sometimes called metal–organic polyhedra, MOPs, or coordination cages). Applications being studied for these materials include gas storage,^3−7^ catalysis,^8−13^ and drug delivery.^14−17^ These applications rely on analysis and control of properties such as size, shape, chemical composition, and reactivity.

Combinations of metal ions and ligands for MOP synthesis are normally chosen with certain target shapes and sizes in mind. For example, Yaghi and co-workers prepared copper-based self-assembled cuboctahedra (MOP-1) and octahedra (MOP-28).^18^ The structure of these MOPs was predicted prior to synthesis and verified by X-ray crystallography.

Some metal–organic materials do not form crystals suitable for X-ray analysis; these can be characterized by alternative analytical techniques. Nuclear magnetic resonance and mass spectrometry have been used extensively to identify MOPs and the intermediates in their formation; examples include the Pt-based molecular cages prepared by Stang et al.^19−23^ and Pd-based molecular complexes by Fujita et al.^24−27^ Scanning probe methods, such as atomic force microscopy (AFM), have been used to investigate MOMs.^28−32^ Kitagawa and co-workers used AFM to characterize coordination star polymers (CSPs) with MOP cores. The CSP and MOP regions could be distinguished by height in the AFM analysis. Additionally, analytical ultracentrifugation (AUC), originally developed to determine the sizes and molecular weights of proteins, has been used to analyze nanoparticles and MOMs.^33−36^ Fujita and co-workers employed AUC to analyze Pd_12_L_24_-type MOPs, with and without ubiquitin guest molecules.^36^

**Figure 1:**
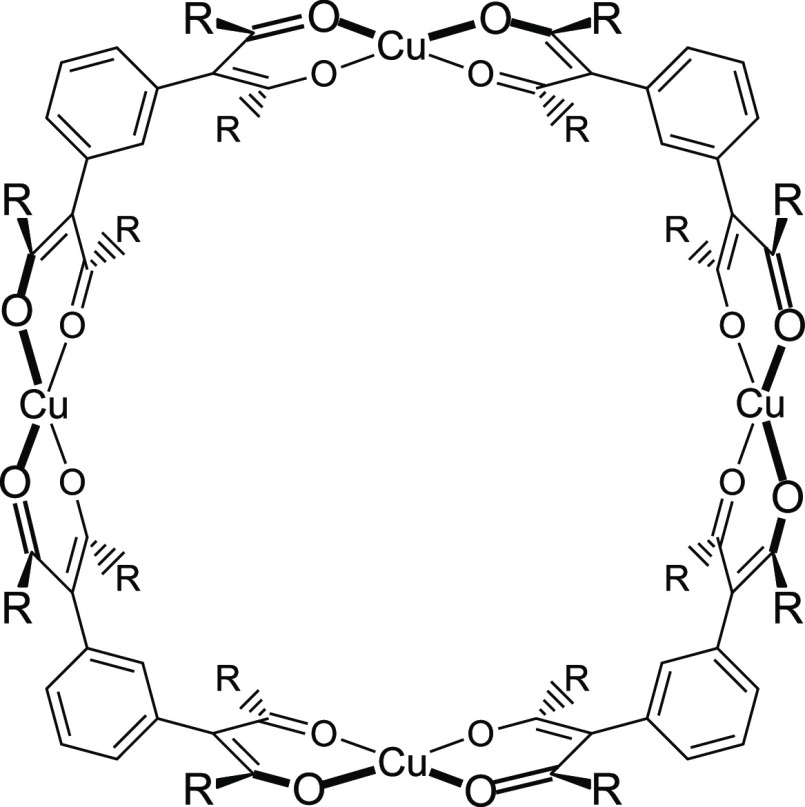
Copper molecular squares prepared from bis(β-diketones). We previously reported Cu_4_(*m*-pba)_4_ (R = CH_3_), Cu_4_(*m*-pbpr)_4_ (R = C_2_H_5_), and Cu_4_(*m*-pbhx)_4_ (R = C_5_H_11_).

Several multifunctional β-diketones and their metal complexes have been prepared. For example, *m*-phenylenebis(β-diketones) react with Cu^2+^ to produce the molecular squares shown in Figure 1. These metal–organic molecules are ca. 1.4 nm in diameter, making them well suited for binding fullerenes (C_60_ and C_70_).^37,38^ Both the empty squares and their host–guest adducts were characterized by X-ray crystallography.

**Figure 2:**
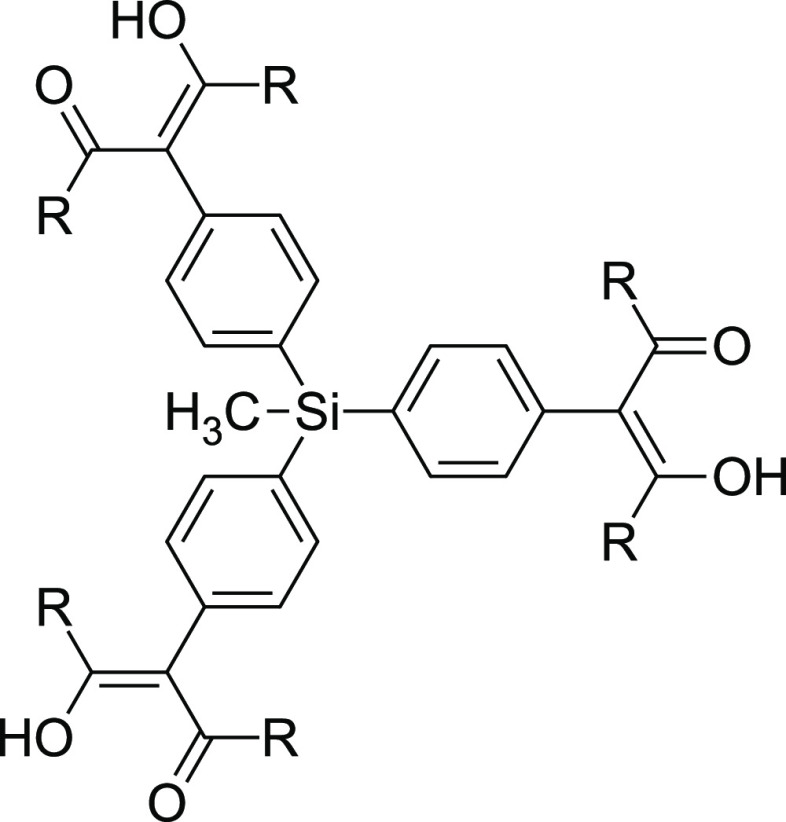
Tris(β-diketones) used in this study: CH_3_Si(phacH)_3_ (R = CH_3_); CH_3_Si(phprH)_3_ (R = C_2_H_5_).

Two organosilicon-based tris(β-diketones), CH_3_Si(phacH)_3_ and CH_3_Si(phprH)_3_ (see Figure 2), are also known.^39^ These ligands coordinate readily to Rh(I) and Ir(I), forming trinuclear complexes, several of whose structures were determined by X-ray crystallography. However, in all of them, each metal atom binds to only one multifunctional ligand; the complexes are not supramolecular.

**Figure 3:**
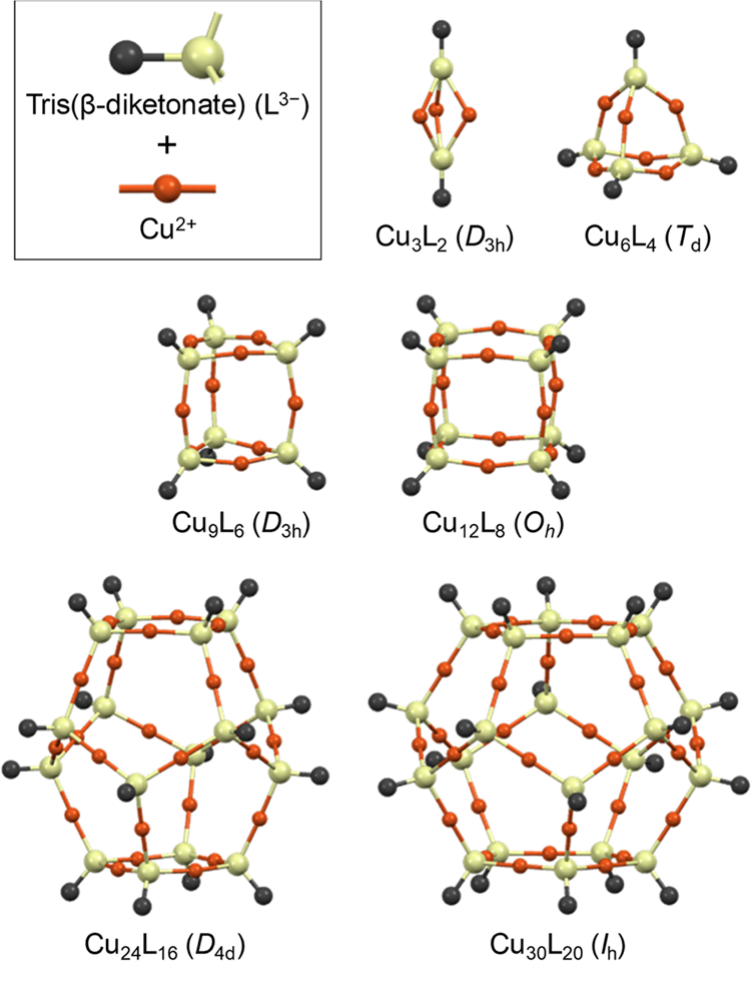
Schematic illustration of metal–organic polyhedra (MOPs) [Cu_3_L_2_]*_n_* that may form from Cu^2+^ (orange spheres) and tris(β-diketonate) ligands L^3–^ (yellow spheres with attached black C atoms). Each MOP is shown with its formula and idealized symmetry point group.

This work concerns new supramolecular complexes prepared by the reaction of these two organosilicon-based tris(β-diketones) with Cu^2+^. The β-diketone moieties in the ligands are approximately at the tetrahedral angle: in our previous X-ray diffraction analyses, the angles between the centroids of the β-diketone moieties averaged 108.5° in the ligands and 109.4° in their Rh complexes.^39^ Cu(II) β-diketonates tend to be approximately planar, making the bonds in the β-diketonate 3 positions approximately collinear. The most likely products of these reactions, based on the combination of linear and trigonal-pyramidal secondary building units (SBUs), are illustrated schematically in Figure 3. A dodecahedral MOP, [Cu_3_L_2_]_10_ or Cu_30_L_20_, permits angles closest to the tetrahedral angle, which should lead to the smallest bond-bending strain and the most stable structure. Smaller MOPs are also possible if the bonds in the Cu-β-diketonate or organosilicon groups are flexible. For example, Li and co-workers reported the self-assembly of supramolecular cubes M_12_L_8_ from trifunctional adamantane-based terpyridine chelating ligands.^40^ The angles between functional groups in these ligands are also close to the tetrahedral angle (ca. 109.5°), so some flexibility is required to produce cube-shaped (90°) and smaller products. Ward and co-workers prepared and characterized MOPs with the formulas M_6_L_9_, M_8_L_12_, and M_16_L_24_ from a single bis(pyridylpyrazole) ligand and showed that the structures can be interconverted in solution.^41^ In our previous work with copper molecular squares (Figure 2), only squares (Cu_4_L_4_) were formed, rather than the hexagons (Cu_6_L_6_) that were expected based on the angle of ca. 120° between the β-diketonates.^37,38^ Thus, β-diketonates are somewhat flexible in the way they bind to Cu^2+^; we have recently shown that metal β-diketonates are among the most flexible among common chelates in their coordination geometry.^42^

The new supramolecular complexes do not form crystals suitable for X-ray analysis; also, their mass spectra do not provide useful information concerning the size of the molecules formed. In this work, we use atomic force microscopy (AFM) and analytical ultracentrifugation (AUC) to provide additional information about the structure of the new supramolecular Cu(II) complexes. The AFM and AUC results suggest that the products are primarily smaller MOPs, i.e., [Cu_3_L_2_]*_n_* with *n* = 1–4.

## Experimental Section

Literature procedures were used to prepare the copper molecular square Cu_4_(m-pbhx)_4_ and the organosilicon-based tris(β-diketones) CH_3_Si(phacH)_3_ and CH_3_Si(phprH)_3_.^37,39^ Solvents and other reagents were obtained from Sigma-Aldrich Co. or other suppliers (HPLC grade when available) and were used without further purification. Molecular modeling was performed by using molecular-mechanics calculations (HyperChem 7.01; Hypercube, Inc., Gainesville, FL); the resulting geometries were similar to those we and others have obtained in X-ray crystal structures of Cu β-diketonate complexes.

### Synthesis of Copper β-Diketonate MOPs

The procedure for [Cu_3_(CH_3_Si(phpr)_3_)_2_]*_n_* is given here; [Cu_3_(CH_3_Si(phac)_3_)_2_]*_n_* was prepared by an analogous method. CuSO_4_·5H_2_O (98 mg, 0.39 mmol) was dissolved in 15 mL of deionized water and converted to [Cu(NH_3_)_4_]^2+^ by treatment with conc. NH_3_(aq) (ca. 1 mL), with stirring. CH_2_Cl_2_ (25 mL) was added to the Cu^2+^ mixture, followed by a solution of CH_3_Si(phprH)_3_ (114 mg, 0.174 mmol) in 75 mL of CH_2_Cl_2_. The mixture was stirred for 6 h at room temperature. The organic layer was transparent and green, indicating the formation of soluble MOPs, and the aqueous layer contained green insoluble material (possibly a polymeric metal–organic product). The aqueous layer was washed three times with 15 mL of CH_2_Cl_2_. The organic layers were combined, dried over Na_2_SO_4_, filtered, and evaporated to dryness. Yield: 94 mg of green powder (72%). Anal. Calcd for C_80_H_90_Cu_3_O_12_Si_2_ (Cu_3_(CH_3_Si(phpr)_3_)_2_): C, 64.47; H, 6.09. Found: C, 64.64; H, 6.00.

### Atomic Force Microscopy

A Keysight 5420 scanning probe microscope (Keysight Technologies, Santa Rosa, CA) with PicoView version 1.12 software was used for scanning probe characterization. Images were acquired using tapping mode under ambient conditions, with a scan rate of 1 line/s. Probe tips were rectangular ultrasharp silicon (Nanoscience Instruments, Phoenix, AZ) with an aluminum reflex coating; the spring constant was 48 N/m, and the average resonance frequency was 176 kHz. Topography and phase images were processed with Gwyddion v. 2.53 open-source software.^43^

The solid Cu MOPs were dissolved in CHCl_3_ or fluorobenzene, and the solutions were drop-deposited on freshly cleaved mica substrates. The solvent was allowed to evaporate in air at 20–25 °C for ca. 2 min, with the substrates in a covered Petri dish to limit deposition of dust or other contaminants. The evaporation of the solvent could be observed visually during this period. Freshly deposited samples were rinsed with ethanol (which does not dissolve the Cu MOPs) and allowed to dry before AFM analysis. For each sample, several images were obtained, and images in which features were well enough separated for height measurement were chosen. In each such image, cursor profiles were recorded for all features that could be resolved clearly.

### Analytical Ultracentrifugation

Equilibrium sedimentation experiments were performed using a Beckman Optima XL-A analytical ultracentrifuge. Cell assemblies contained sapphire windows and aluminum double-sector centerpieces with a 12 mm path length. They were inserted into a four-hole rotor and allowed to cool to 20 °C while under vacuum. Sedimentation equilibrium was attained at rotation speeds of 40 000–60 000 rpm over 24–48 h. No further change in the absorption profile occurred over an additional 24 h period. Data were recorded at wavelengths between 315 and 350 nm, and analyzed by means of the kDALTON program^44^ or via standard linear or nonlinear least-squares routines.

## Results and Discussion

**Figure 4:**
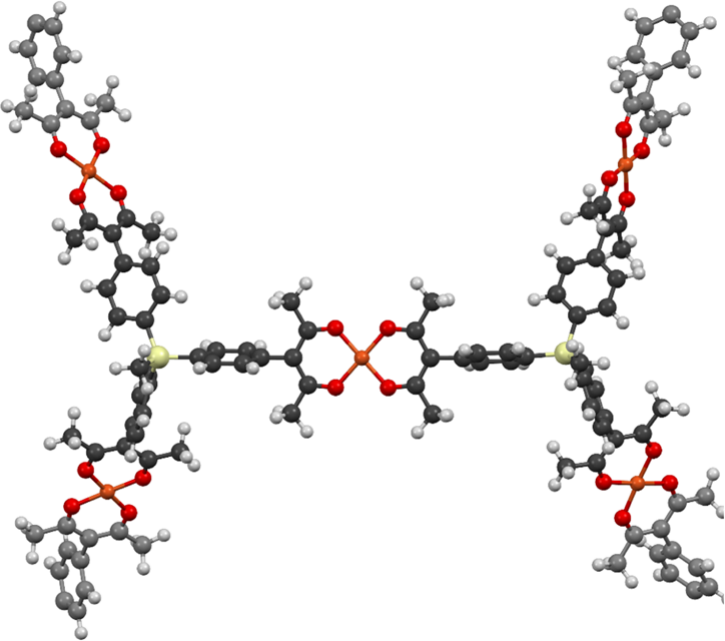
Molecular model of a portion of the proposed general MOP structure derived from Cu^2+^ and tris(β-diketone) CH_3_Si(phacH)_3_, showing square-planar Cu(β-diketonate)_2_ and trigonal-pyramidal tris(β-diketonate) moieties.

The tris(β-diketones) used to construct MOPs in this study appear in Figure 2. Reaction of each ligand (in a CH_2_Cl_2_ solution) with [Cu(NH_3_)_4_^2+^](aq) produces a green solid that is soluble in nonpolar solvents. We propose that these materials consist of metal–organic polyhedra (MOPs) with the general formula [Cu_3_L_2_]*_n_* (LH_3_ = CH_3_Si(phacH)_3_ or CH_3_Si(phprH)_3_). A portion of the proposed structure is shown in Figure 4: each Cu atom is bound to two β-diketonates, and each ligand is bound to three Cu atoms.

**Figure 5:**
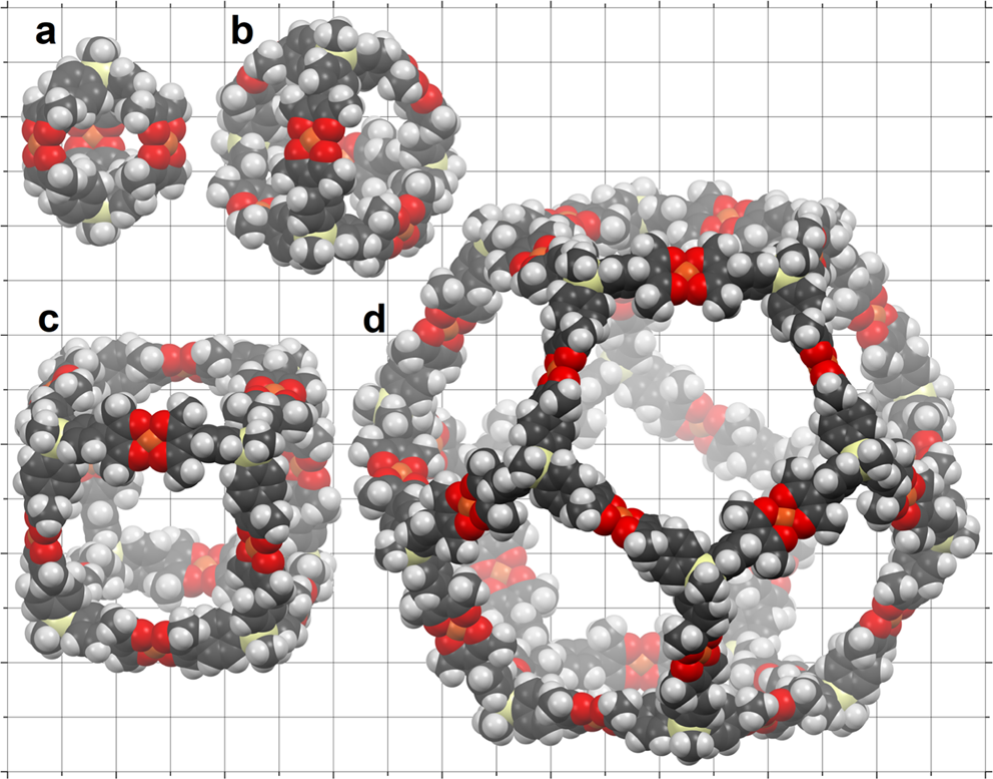
Space-filling models of four possible MOPs [Cu_3_(CH_3_Si(phac)_3_)_2_]*_n_*: (a) the smallest possible MOP, *n* = 1, i.e., Cu_3_L_2_; (b) a tetrahedral MOP, *n* = 2, i.e., Cu_6_L_4_; (c) a cubic MOP, *n* = 4, i.e., Cu_12_L_8_; and (d) a dodecahedral MOP, *n* = 10, i.e., Cu_30_L_20_. The models are superimposed on a 0.5 nm grid. Coordinates for the models were derived from molecular-mechanics calculations.

Six possible products of the synthesis are shown schematically in Figure 3: [Cu_3_L_2_]*_n_*, where *n* = 1, 2, 3, 4, 8, and 10. In Figure 5, four of these (*n* = 1, 2, 4, and 10) are shown as space-filling models; their estimated sizes range from 1 to 5 nm.

Microanalytical data for the new compounds support the empirical formula of Cu_3_L_2_. However, in contrast to the supramolecular Cu β-diketonates we have reported previously, the new MOPs do not crystallize, so their structures cannot be determined directly via X-ray crystallography. (We attempted to grow crystals of the compounds by solvent layering and vapor diffusion, techniques that have been successful in our previous work, but no crystals were observed in the products. Powder diffraction is not appropriate for these materials: due to the limited amount of data it produces, it is best suited for determining the arrangement of known molecules in crystals, rather than identification of unknown molecules.^45,46^) Mass spectra (MALDI, ESI) of the new compounds did not reveal useful parent ion signals. The compounds are green in the solid state and in solution. However, all of the likely products, [Cu_3_L_2_]*_n_* (*n* = 1–10), contain the same components in the same proportions and approximately the same geometry (see Figures 4 and 5). Thus, their UV–visible extinction coefficients are expected to be approximately the same per [Cu_3_L_2_] unit. (We have observed this previously in studies of Cu β-diketonates containing 1–4 Cu atoms). Therefore, in this work, we studied the MOPs by atomic force microscopy and analytical ultracentrifugation, which are capable of distinguishing molecules of different sizes.

### Atomic Force Microscopy (AFM)

Samples of [Cu_3_(CH_3_Si(phac)_3_)_2_]*_n_* and [Cu_3_(CH_3_Si(phpr)_3_)_2_]*_n_* prepared on mica substrates were analyzed by tapping-mode AFM. For each sample, scans of several different areas were performed, and the heights of all detected features in each scan were measured. A total of 263 height measurements were made for [Cu_3_(CH_3_Si(phac)_3_)_2_]*_n_* and 179 for [Cu_3_(CH_3_Si(phpr)_3_)_2_]*_n_*.

**Figure 6:**
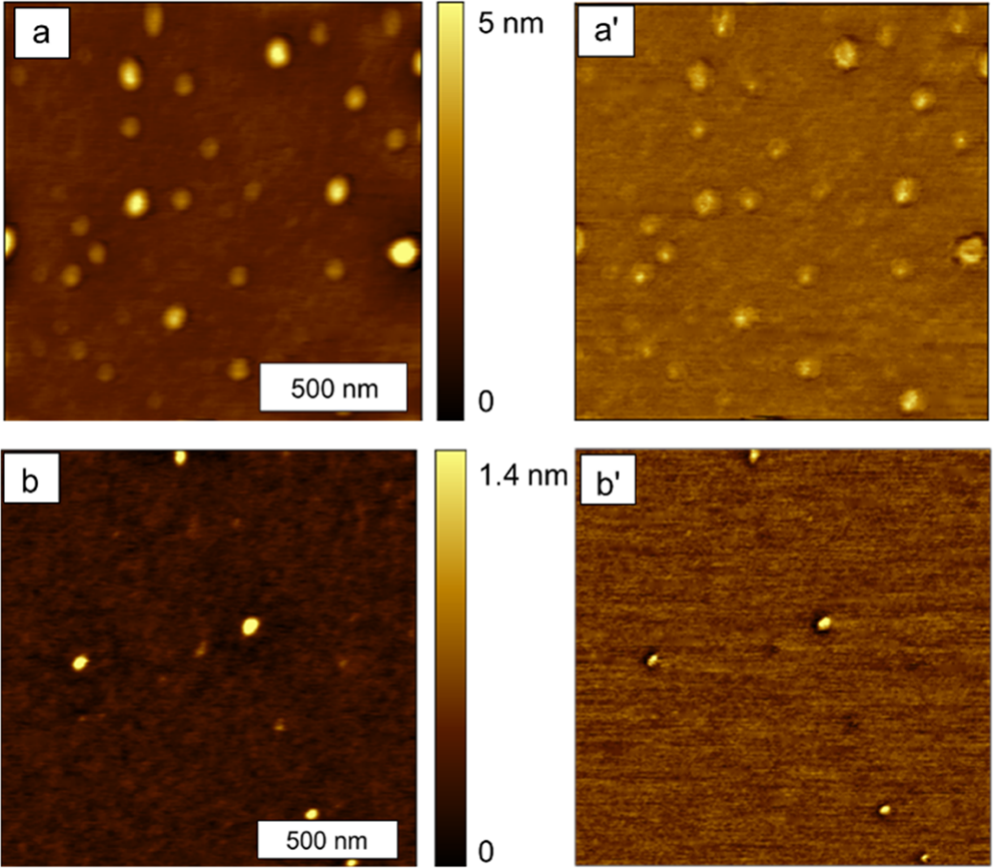
AFM images of samples prepared on mica. (a) Topography image of [Cu_3_(CH_3_Si(phac)_3_)_2_]*_n_*; (a′) simultaneously acquired phase image; (b) topography image of [Cu_3_(CH_3_Si(phpr)_3_)_2_]*_n_*; (b′) phase image. Images are 1.5 × 1.5 μm^2^ and were acquired in ambient air in tapping mode.

**Figure 7:**
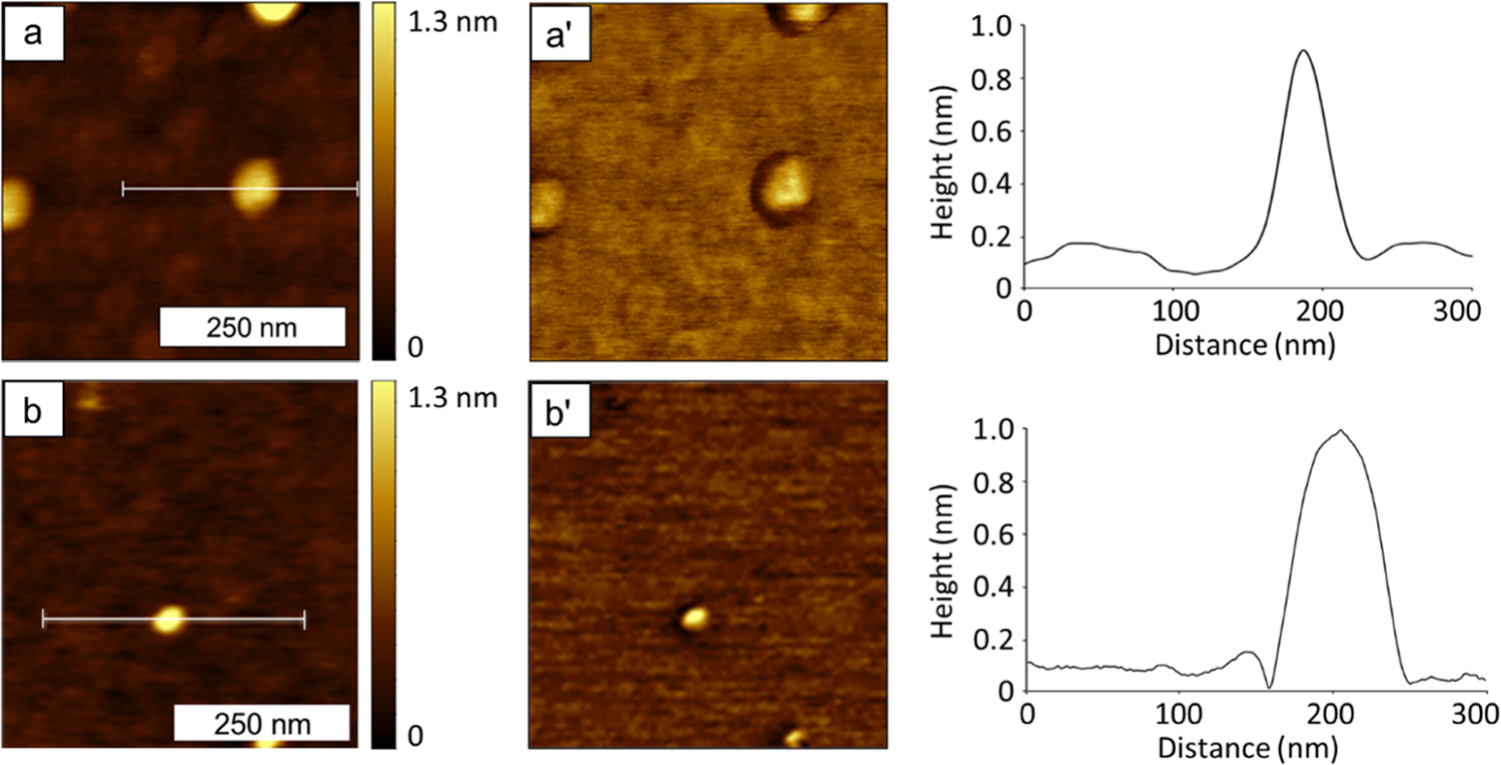
Individual molecules of samples viewed with AFM. (a) Topography and (a′) phase image of [Cu_3_(CH_3_Si(phac)_3_)_2_]*_n_*; (b) topography and (b′) phase image of [Cu_3_(CH_3_Si(phpr)_3_)_2_]*_n_*; image sizes are 500 × 500 nm^2^. A cursor profile for each molecule is shown on the right.

Selected topography and phase frames for the two samples (prepared on freshly cleaved mica substrates) are presented in Figure 6. Higher-resolution images and example cursor profiles are listed in Figure 7. Additional AFM images of both compounds are available in the Supporting Information.

**Figure 8:**
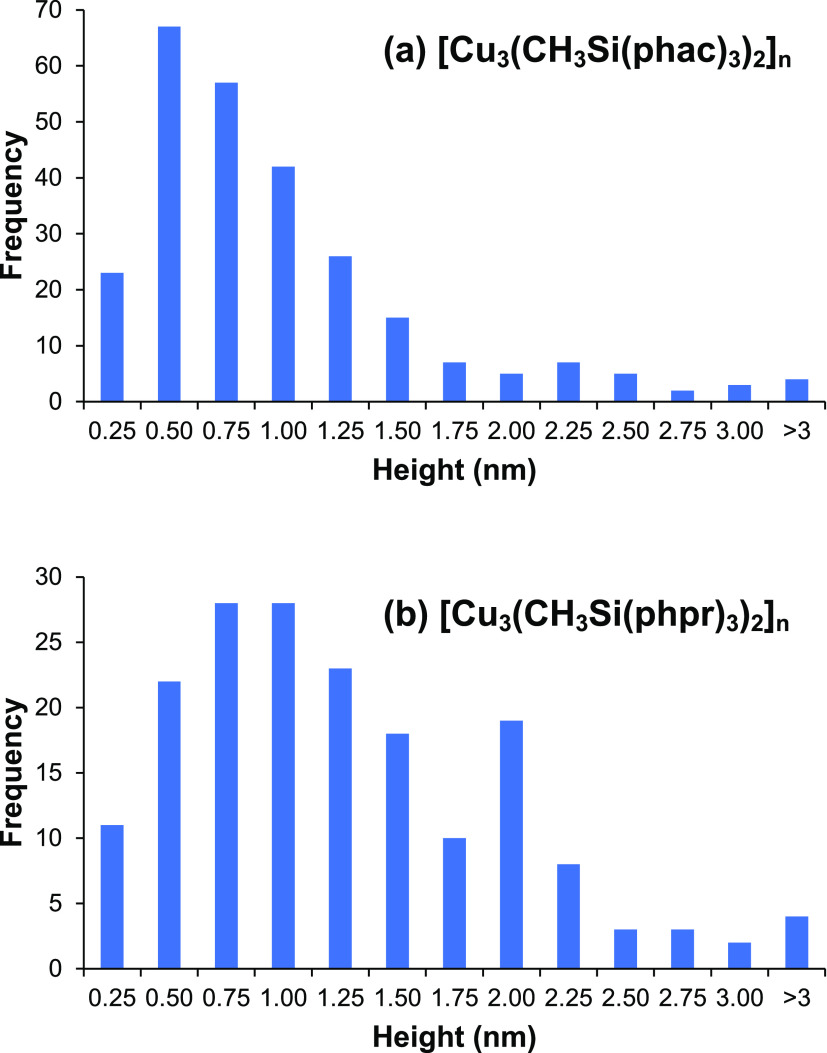
Histograms showing the heights of features observed in AFM measurements. (For example, “0.75 nm” means heights >0.5 and ≤0.75 nm).

The feature heights observed in the AFM measurements have averages and standard deviations of 0.88 ± 0.67 nm for [Cu_3_(CH_3_Si(phac)_3_)_2_]*_n_* and 1.16 ± 0.73 nm for [Cu_3_(CH_3_Si(phpr)_3_)_2_]*_n_*. Histograms of the feature heights are shown in Figure 8. These values can be compared to approximate heights of 1.0 nm for [Cu_3_(CH_3_Si(phac)_3_)_2_] (i.e., *n* = 1), and 2.5 nm for the molecular cube, [Cu_3_(CH_3_Si(phac)_3_)_2_]_4_ (*n* = 4); see Figure 5. (The histograms can also be fitted to normal distributions, with μ = 0.58 and 0.41 nm for [Cu_3_(CH_3_Si(phac)_3_)_2_]*_n_*, and μ = 0.89 and 0.77 nm for [Cu_3_(CH_3_Si(phpr)_3_)_2_]*_n_*. However, these values should be interpreted with caution, because the distributions are clearly not symmetrical, likely reflecting the fact that the samples contain molecules of several different sizes).

For [Cu_3_(CH_3_Si(phac)_3_)_2_]*_n_*, most features have heights less than 2 nm, but for [Cu_3_(CH_3_Si(phpr)_3_)_2_]*_n_* proportionately, more tall features are found. The CH_3_Si(phprH)_3_ ligand is larger because of the ethyl groups in its β-diketone moieties. However, the ethyl groups are flexible and may not add much to the overall molecular height, as measured in AFM. The taller features may be associated with larger polyhedra, or they may result from the aggregation of several smaller molecules.

To summarize, we have applied AFM to identify the likely composition of the new MOPs [Cu_3_L_2_]*_n_*. In contrast, in the applications of AFM to supramolecular metal–organic materials mentioned above,^28−32^ the structure of the material was known, and AFM was used to study its behavior when adsorbed on a substrate.

### Analytical Ultracentrifugation (AUC)

We performed two sets of equilibrium sedimentation experiments. First, we measured the AUC concentration (absorption) profile of the molecular square Cu_4_(*m*-pbhx)_4_ (see Figure 1; R = C_5_H_11_), a supramolecular copper β-diketonate complex of known structure. Then, we made similar measurements of the unknown copper complexes [Cu_3_(CH_3_Si(phac)_3_)_2_]*_n_* and [Cu_3_(CH_3_Si(phpr)_3_)_2_]*_n_*.

### Equilibrium AUC of Cu_4_(*m*-pbhx)_4_

**Figure 9:**
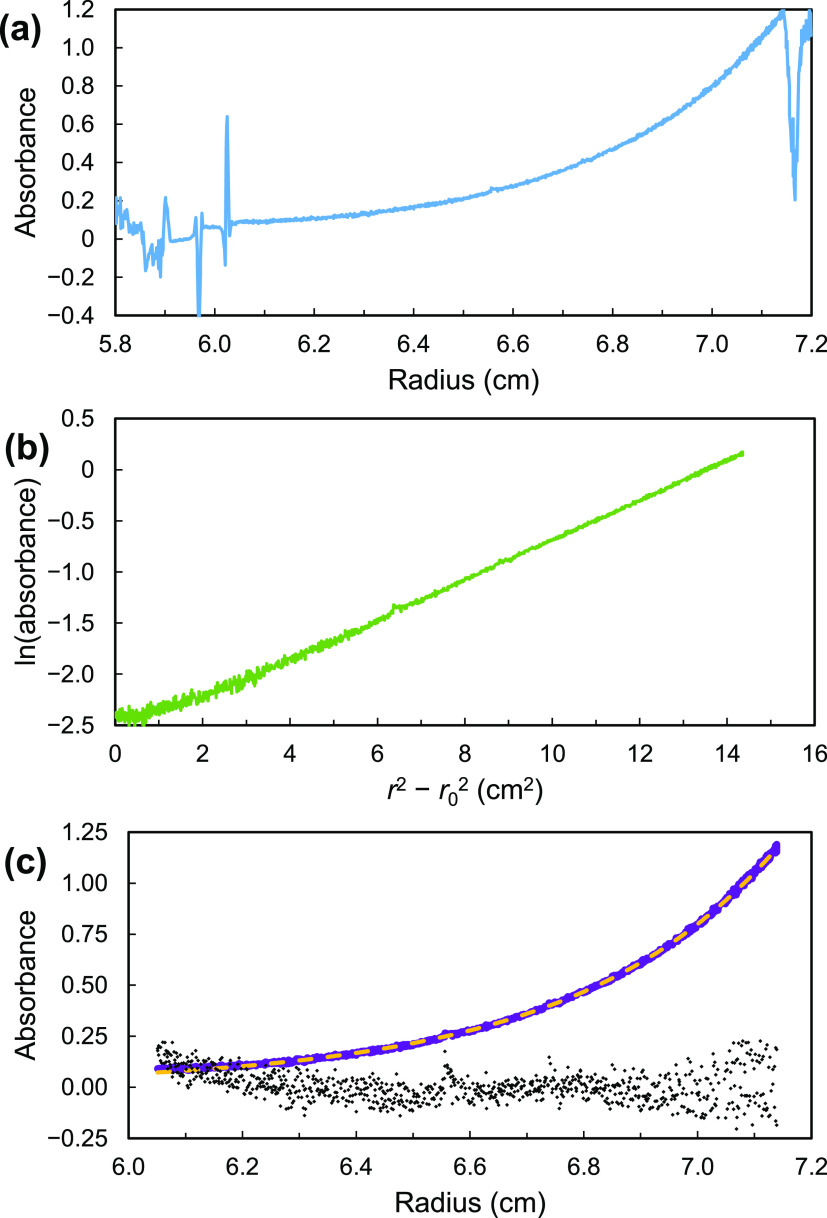
Equilibrium sedimentation of Cu_4_(*m*-pbhx)_4_ in toluene solution at 293 K and 40 000 rpm. (a) All data. (b) Plot of ln(*A*) vs *r*^2^ – *r*_0_^2^ (*r*_0_ = 6.05 cm). (c) Experimental data (purple); nonlinear fit to eq 1 (yellow); and residuals (×10, black). In (b) and (c), only data between 6.05 and 7.12 cm are plotted.

This molecular square (*M*_W_ = 2241.08 g/mol) is soluble and stable in a variety of solvents.^37^ Sedimentation of Cu_4_(*m*-pbhx)_4_ was measured in the following four solvents: toluene, toluene-*d*_8_, tetralin, and fluorobenzene (ρ = 0.867, 0.945, 0.970, and 1.022 g/mL, respectively, at 20 °C). Figure 9 shows the behavior of Cu_4_(*m*-pbhx)_4_ in one experiment in a toluene solution.

In equilibrium AUC, the absorbance of the sample at a particular radial distance, *A*(*r*), represents the analyte concentration through the Beer–Lambert law. The absorbance profile is given^47^ by
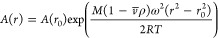
1where *r*_0_ is a reference point (often a position close to the meniscus, or the first or last data point used in fitting); *M* and *v̅* are the molecular weight and partial specific volume of the analyte; ρ is the density of the solvent; and ω is the angular speed of the centrifuge.

For the molecular square Cu_4_(*m*-pbhx)_4_, *M* is known, so its partial specific volume *v̅* can be determined from the sedimentation curve, via the slope of a plot of ln(*A*) vs *r*^2^ – *r*_0_^2^ (see Figure 9b), or by a nonlinear fit to eq 1 above (see Figure 9c). In these experiments, both approaches gave good results. The values of *v̅* obtained from nonlinear fits in these experiments were 0.889, 0.892, 0.850, and 0.902 cm^3^ g^–1^ (in toluene, toluene-*d*_8_, tetralin, and fluorobenzene, respectively), for an average of 0.886 ± 0.019 cm^3^ g^–1^.

Durchschlag and Zipper (referred to below as “D–Z”)^48^ described an empirical method for estimating the partial specific volume of organic compounds. Their method yields an estimate of 0.839 cm^3^ g^–1^ for Cu_4_(*m*-pbhx)_4_ (see the Supporting Information for details), which differs by about 5% from the experimental value.

D–Z reported that their method generally produced partial specific volumes that were within ∼2% of the experimental values. The discrepancy for Cu_4_(*m*-pbhx)_4_ is somewhat larger. This could be due to the different kind of material we are studying (a nonpolar compound dissolved in organic solvents, whereas D–Z’s data are for polar and ionic solutes in water). Also, the somewhat higher experimental value of *v̅* may reflect inefficient packing of solvent molecules around and within supramolecular structures.

Two conclusions can be drawn from these AUC measurements on the known Cu_4_(*m*-pbhx)_4_ molecular square. First, it behaves like an ideal noninteracting solute in four nonpolar organic solvents. Second, its experimental partial specific volume, *v̅*, is close to that estimated by the D–Z method. [Cu_3_(CH_3_Si(phac)_3_)_2_]*_n_* and [Cu_3_(CH_3_Si(phpr)_3_)_2_]*_n_* are chemically similar to Cu_4_(*m*-pbhx)_4_, so the D–Z method is likely to give a useful estimate of their partial specific volumes as well.

### Equilibrium AUC of [Cu_3_(CH_3_Si(phac)_3_)_2_]*_n_* and [Cu_3_(CH_3_Si(phpr)_3_)_2_]*_n_*

At first, we prepared solutions of these new materials in CH_2_Cl_2_ and CHCl_3_, because we previously studied supramolecular copper β-diketonates in these solvents. However, the solutions showed signs of decomposition (loss of absorbance, and formation of gel-like solids) within 24–48 h. Although such solutions may be suitable for quick reactions or spectral measurements, they are not stable enough for equilibrium AUC studies. We found that solutions in chlorobenzene were much more stable, but AUC measurements on these solutions showed slow sedimentation.

This observation, along with the AUC results described above for Cu_4_(*m*-pbhx)_4_, suggested that the partial specific volume of the new Cu compounds is relatively high, meaning that sedimentation would occur fast enough to avoid decomposition only in a solvent of lower density than chlorobenzene (ρ = 1.11 g cm^–3^). Trial experiments indicated that solutions of [Cu_3_(CH_3_Si(phac)_3_)_2_]*_n_* and [Cu_3_(CH_3_Si(phpr)_3_)_2_]*_n_* in toluene and fluorobenzene were stable for at least 72 h and also showed measurable sedimentation.

**Figure 10:**
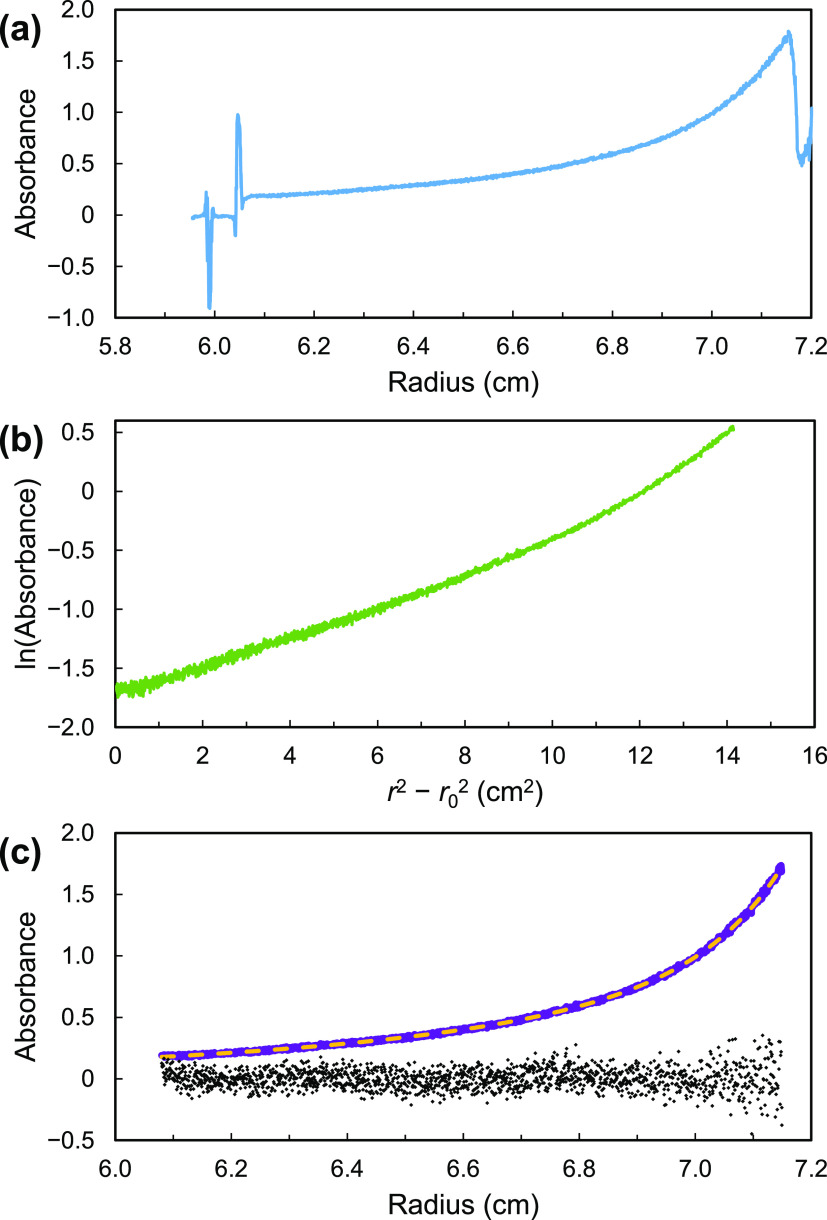
Equilibrium sedimentation of [Cu_3_(CH_3_Si(phac)_3_)_2_]*_n_* in fluorobenzene solution at 293 K and 60 000 rpm. (a) All data. (b) ln(*A*) vs *r*^2^ – *r*_0_^2^ (*r*_0_ = 6.08 cm). (c) Experimental data (purple); biexponential nonlinear fit to eq 2 (yellow); and residuals (×10, black). In parts (b) and (c), only data between 6.08 and 7.15 cm are plotted.

Equilibrium sedimentation experiments were performed on [Cu_3_(CH_3_Si(phac)_3_)_2_]*_n_* and [Cu_3_(CH_3_Si(phpr)_3_)_2_]*_n_* in toluene and fluorobenzene. The results for one experiment with [Cu_3_(CH_3_Si(phac)_3_)_2_]*_n_* in fluorobenzene are shown in Figure 10. The logarithmic plot in Figure 10b is noticeably curved, indicating that this material does not consist of a single pure compound.

If the samples consist of MOPs of two different sizes, the AUC data should show biexponential behavior
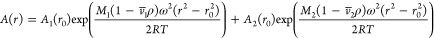
2where subscripts 1 and 2 refer to the two different MOPs in the sample. As shown in Figure 10c, the AUC data fit eq 2 well; this was true for all of our AUC experiments with [Cu_3_(CH_3_Si(phac)_3_)_2_]*_n_* and [Cu_3_(CH_3_Si(phpr)_3_)_2_]*_n_*.

In order to estimate the molecular weights *M*_1_ and *M*_2_ for the two components in this sample, we needed estimates of their partial specific volumes. The D–Z method (discussed above) gave the following estimates of *v̅*: 0.716 ([Cu_3_(CH_3_Si(phac)_3_)_2_]*_n_*) and 0.771 cm^3^ g^–1^ ([Cu_3_(CH_3_Si(phpr)_3_)_2_]*_n_*). (These correspond to densities of ca. 1.40 and 1.30 g cm^–3^, respectively, consistent with the need for low-density solvents for observable sedimentation before sample decomposition occurs). If the molecules in our sample are MOPs with two different sizes, e.g., [Cu_3_(CH_3_Si(phac)_3_)_2_]*_n_* with different *n* values, the D–Z method will yield the same *v̅* value. This is a good first approximation because each member of the proposed family of MOPs (Figure 5) is made up of chemically identical pieces in the same proportions. The resulting values of *M*_1_ and *M*_2_ are shown in Table 1. (Additional discussion of *v̅* for MOPs of different sizes is in the Supporting Information).

**Table 1:** Calculated Molecular Weights of the Two Components in [Cu_3_(CH_3_Si(phac)_3_)_2_]*_n_* and [Cu_3_(CH_3_Si(phpr)_3_)_2_]*_n_*, Based on AUC Equilibrium Sedimentation Data

sample	solvent	*M*_1_ (g mol^–1^)^a^	*M*_2_ (g mol^–1^)^a^
[Cu_3_(CH_3_Si(phac)_3_)_2_]*_n_*	fluorobenzene	550	2400
[Cu_3_(CH_3_Si(phac)_3_)_2_]*_n_*	toluene	400	1800
[Cu_3_(CH_3_Si(phpr)_3_)_2_]*_n_*	fluorobenzene	750	4000
[Cu_3_(CH_3_Si(phpr)_3_)_2_]*_n_*	toluene	880	3400

a Using these estimated partial specific volumes: [Cu_3_(CH_3_Si(phac)_3_)_2_]*_n_*, 0.716 cm^3^ g^–1^; [Cu_3_(CH_3_Si(phpr)_3_)_2_]*_n_*, 0.771 cm^3^ g^–1^. Standard errors in the fitted *M*_1_ and *M*_2_ values were 1–7% in each experiment.

*M*_1_ and *M*_2_ should be multiples of the Cu_3_L_2_ formula weight (1322.05 g mol^–1^ for Cu_3_(CH_3_Si(phac)_3_)_2_, or 1490.37 g mol^–1^ for Cu_3_(CH_3_Si(phpr)_3_)_2_). However, the values in Table 1 are not very close to these. Also, although the data for individual sedimentation experiments are very well fit by the equation for two independent solutes, the results are not highly reproducible from one run to the next (for the same solute–solvent combination) or between fluorobenzene and toluene.

We explored whether molecules having Cu:L ratios other than 3:2 might agree better with the *M* values in Table 1. For example, the *M*_1_ values for the Cu-CH_3_Si(phpr)_3_ complex are closer to that for a 1:1 complex, Cu(CH_3_Si(phpr)_2_(phprH)) (714.4 g mol^–1^). Such a molecule would require a highly strained conformation for two of its β-diketonate groups to chelate to the Cu atom; it would not agree with the observed microanalytical data; and it would also require that not all β-diketonates be coordinated to Cu. In our previous work, we prepared a variety of supramolecular metal complexes of multifunctional β-diketonates, and whenever we isolated a product that is cleanly soluble and stable in an organic solvent, all of its metal and β-diketonate valences have been filled. Thus, it is not clear that there is a chemically reasonable alternative to the [Cu_3_L_2_]*_n_* formulas.

In the biexponential model of eq 2, the fitted values of *A*_1_ and *A*_2_ indicate the contribution of each component to the absorbance. In all of our experiments, *A*_1_ > *A*_2_, meaning that the majority of light absorption is due to the lighter component 1. Also, [Cu_3_L_2_]*_n_* molecules with larger values of *n*, containing more of the same constituents, should absorb light more strongly. Thus, the mixtures are likely to consist primarily (>90%) of the lighter component.

In addition to AFM and AUC measurements, dynamic light scattering (DLS) analysis was attempted on a sample of [Cu_3_(CH_3_Si(phac)_3_)_2_]*_n_*. Dynamic light scattering is often used to analyze higher-molecular-weight materials, such as proteins and nanoparticles.^49^ For example, in a recent report, MOPs with diameter ca. 3 nm were polymerized to produce larger particles (50–180 nm), and this process was followed by DLS.^50^ For the compounds studied here, the species with the highest molecular weights, [Cu_3_L_2_]*_n_* (*n* = 8, 10), are, in principle, large enough to produce DLS signals. We prepared a solution of [Cu_3_(CH_3_Si(phac)_3_)_2_]*_n_* in CH_2_Cl_2_ and attempted to measure autocorrelation functions by using laser wavelengths of 488.0, 514.5, 632.8, and 647.0 nm. The green solutions of [Cu_3_(CH_3_Si(phac)_3_)_2_]*_n_* absorbed light at all four laser wavelengths, confounding our attempts by reducing the incident light reaching the center of the 13 mm diameter scattering cells and the scattered light emanating from that point. While it might prove possible to measure DLS signals in cells with smaller path lengths, it was more expedient to take advantage of the absorbance by selecting the AUC experiment over DLS.

## Conclusions

In this work, atomic force microscopy (AFM) and analytical ultracentrifugation (AUC) were used to probe the structures of two supramolecular copper β-diketonate metal–organic polyhedra (MOPs), [Cu_3_(CH_3_Si(phac)_3_)_2_]*_n_* and [Cu_3_(CH_3_Si(phpr)_3_)_2_]*_n_*, when more conventional molecular characterization methods failed. This is an unusual approach because previous applications of AFM and AUC to supramolecular metal–organic systems have been to species whose structures were already known. AFM experiments on the new MOPs revealed features with heights in the range of 0.5–3 nm. These features are likely to represent individual molecules [Cu_3_L_2_]*_n_* (*n* = 1–4). AUC equilibrium experiments on the new [Cu_3_L_2_]*_n_* MOPs indicated that each sample consisted of lighter and heavier components, with the lighter component dominant and with *M*_2_/*M*_1_ = 4–6. Although the calculated molecular weights showed only qualitative agreement with the [Cu_3_L_2_]*_n_* formula, the results indicate overall that these MOPs are nanoscale 3D molecules with molecular weights in the 500–5000 range. Nanoscale measurements with the complementary techniques of AFM and AUC are promising for studying supramolecular systems of unknown structure. Future studies by these methods may reveal the reasons for some of the discrepancies we found in our work and may stimulate comparison with other analytical methods.

## Supporting Information Available

Additional AFM images of [Cu_3_(CH_3_Si(phac)_3_)_2_]*_n_*, and [Cu_3_(CH_3_Si(phpr)_3_)_2_]*_n_*; estimation of partial specific volume *v̅* for Cu_4_(*m*-pbhx)_4_, [Cu_3_(CH_3_Si(phac)_3_)_2_]*_n_*, and [Cu_3_(CH_3_Si(phpr)_3_)_2_]*_n_*, by the Durchschlag–Zipper (D–Z)^48^ method; and possible effects of MOP size on *v̅* (7 pp) (PDF)
