# Supplementary material for: Structural Information on Supramolecular Copper(II) β-Diketonate Complexes from Atomic Force Microscopy and Analytical Ultracentrifugation
Source: ACS Omega. 2024 Jan 4;9(2):2629–38. doi: 10.1021/acsomega.3c07493 (PMC10795041; doi:10.1021/acsomega.3c07493)
Supplement: Supplementary file 1 — ao3c07493_si_001.pdf [file ao3c07493_si_001.pdf]

Structural Information on Supramolecular Copper(II) β-Diketonate Complexes from Atomic Force Microscopy and Analytical Ultracentrifugation

Jonathan S. Casey, Ashley R. Walker, Xianglin Zhai, Jayne C. Garno, Paul S. Russo, and Andrew W. Maverick*

Department of Chemistry and Macromolecular Studies Group, Louisiana State University, Baton Rouge, LA 70803, United States

Electronic Supporting Information

Contents

A. Additional AFM Images S2-S3

B. Partial Specific Volumes for the Metal-Organic Materials S4-S7

A. Additional AFM Images

1. Compound 1, $[Cu_3(CH_3Si(phac)_3)_2]_n$

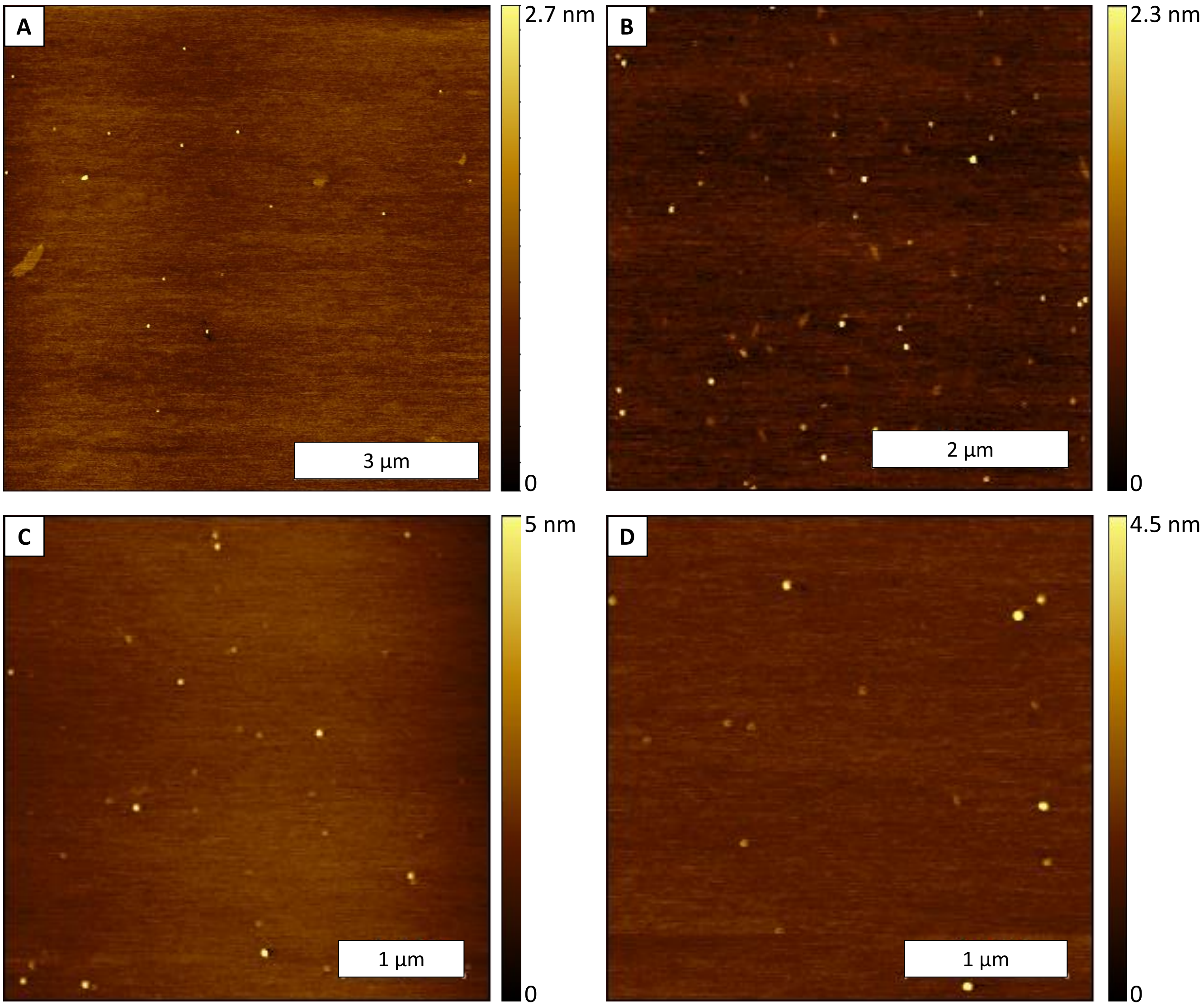

Figure S1. Additional AFM images of $[Cu_3(CH_3Si(phac)_3)_2]_n$ prepared on mica, captured in tapping mode. Topography frames: [A] 8×8 µm²; [B] 5×5 µm²; [C] 4×4 µm²; [D] 3×3 µm².

2. Compound 2, $[Cu_3(CH_3Si(phpr)_3)_2]_n$

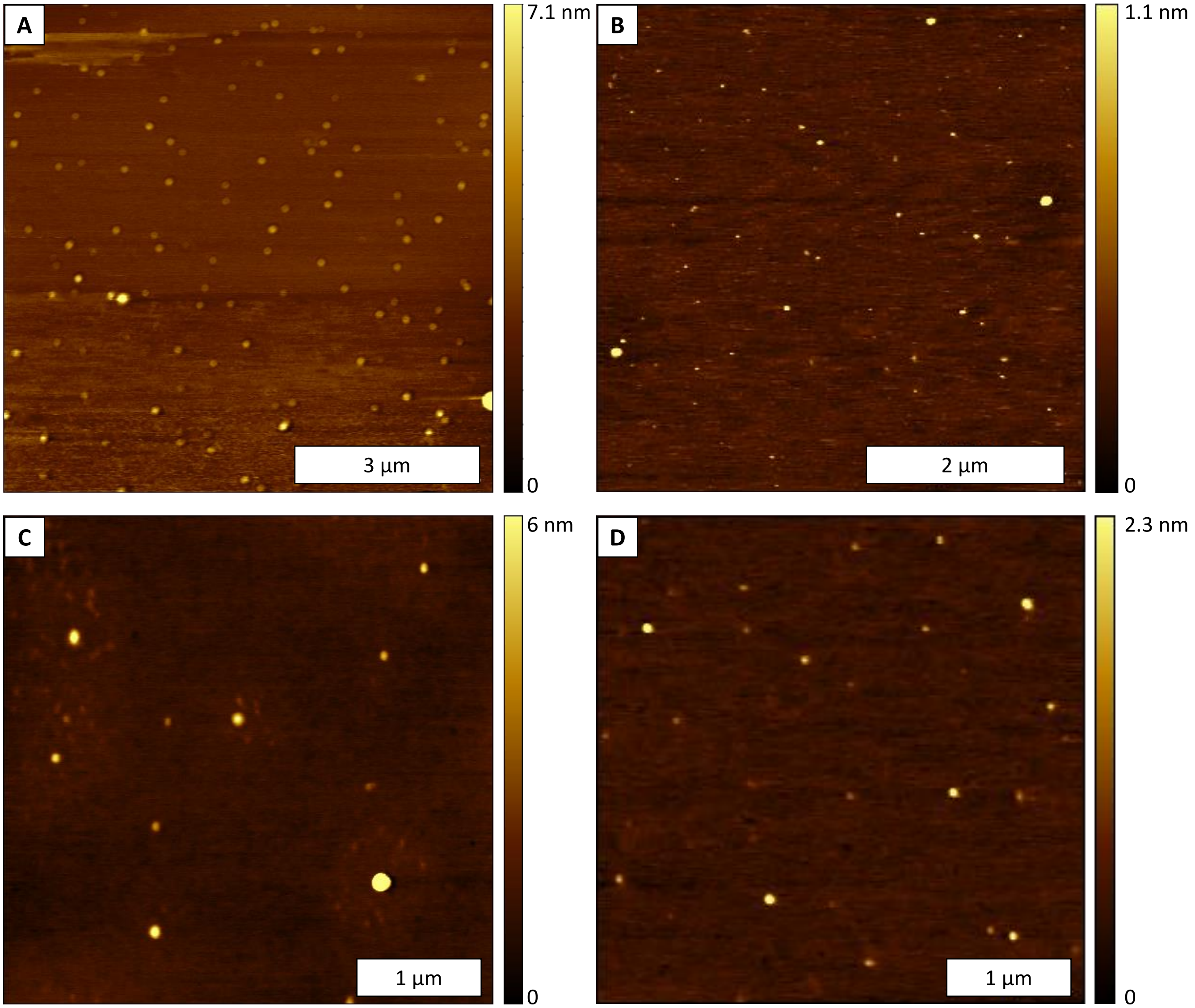

Figure S2. Additional AFM images of $[Cu_3(CH_3Si(phpr)_3)_2]_n$ prepared on mica, captured in tapping mode. Topography frames: [A] 8×8 µm²; [B] 5×5 µm²; [C] 4×4 µm²; [D] 4×4 µm².

B. Partial Specific Volumes for the Metal-Organic Materials

1. Durchschlag-Zipper (D-Z) estimates

Durchschlag and Zipper (D-Z) developed an empirical procedure for estimating partial specific volumes, based on data for organic compounds (including small molecules and proteins) in aqueous solution.[S1] Our AUC experiments with the known Cu_4(*m*-pbhx)$_4$ molecular square gave us a reference point for a compound that is similar in structure and solubility to the new MOPs, $[Cu_3(CH_3Si(phac)_3)_2]_n$ and $[Cu_3(CH_3Si(phpr)_3)_2]_n$. We first compared the experimental partial specific volume for Cu_4(*m*-pbhx)$_4$ to that estimated by the D-Z method. This allowed us to use the D-Z procedure to obtain an estimate of $\bar{v}$ for the new MOPs, which permits estimating their molecular weights.

The D-Z formula for the partial molar volume $\bar{V}_c$ is

$$\bar{V}_c = \sum V_i + V_{CV} - \sum V_{RF} - \sum V_{ES} \quad \text{(eq S1)}$$

where the V_i are increments for individual atoms and functional groups, V_{CV} is a covolume correction of +12.4 cm^3 mol^{-1}, the V_{RF} are corrections for ring formation, and the V_{ES} are electrostriction corrections (which are zero for the neutral MOPs discussed here).

We used the following increments V_i, from ref S2: C, 9.9 cm^3 mol^{-1}; H, 3.1 cm^3 mol^{-1}; O (ketone), 5.5 cm^3 mol^{-1}; and Cu: 7.1 cm^3 mol^{-1}.

The D-Z papers do not give a value for Si. Our estimate (26.1 cm^3 mol^{-1}) was obtained by comparing experimental densities of three simple liquid organosilicon compounds (tetramethylsilane, 0.648 g cm^{-3}; triethylsilane, 0.729 g cm^{-3}; and phenyltrimethylsilane, 0.873 g cm^{-3}) with those of their all-carbon analogs (0.601, 0.694, and 0.867 g cm^{-3}, respectively). Replacing a C atom by Si in these compounds increases the molar volume by 15-17 cm^3. The

average of the three increases is 16.2 cm^3 mol^{-1}, which gives a D-Z value of 26.1 cm^3 mol^{-1} for Si.

Note: Changing the volume increment for Si by ±2 cm^3 mol^{-1} leads to changes in estimated $\bar{v}$ values of <0.01 cm^3 g^{-1} for the three reference compounds. The new Cu-MeSi MOPs have an even smaller fraction of Si, so any errors in the volume increment for Si probably do not affect the calculated $\bar{v}$ values for our MOPs significantly.

Assignments of ring corrections in the D-Z formula were made as follows: A Cu(β-diketonate)$_2$ moiety contains two 6-membered rings (V_{RF} = 8.1 cm^3 mol^{-1}). The molecular square Cu_4(*m*-pbhx)$_4$ contains one large ring (V_{RF} = 14.1 cm^3 mol^{-1}), and the Cu-Si MOPs contain three large rings per Cu_3L_2 unit.

Results for the Cu_4 molecular square and the two MOPs are shown below.

**Cu_4(*m*-pbhx)$_4$ square, $C_{128}H_{192}Cu_4O_{16}$ (*M* = 2241.06 g mol^{-1}):**<br>$\bar{V}_c$ = {(128 × 9.1) + (192 × 3.1) + (4 × 7.1) + (16 × 5.5)} + 12.4 − (12 × 8.1) − 14.1<br>= 1879.9 cm^3 mol^{-1}<br>Estimated density = 1.192 g cm^{-3}; estimated partial specific volume $\bar{v}$ = 0.839 cm^3 g^{-1}.
**Cu_3(MeSi(phac)$_3$)$_2$ MOP, $C_{68}H_{66}Cu_3O_{12}Si_2$ for n = 1 (*M* = 1322.05 g mol^{-1}):**<br>$\bar{V}_c$ = {(68 × 9.1) + (66 × 3.1) + (3 × 7.1) + (12 × 5.5) + (2 × 26.1)} + 12.4 − (12 × 8.1) − (3 × 14.1) = 890.2 cm^3 mol^{-1}<br>Estimated density = 1.485 g cm^{-3}; estimated partial specific volume $\bar{v}$ = 0.673 cm^3 g^{-1}.
**Cu_3(MeSi(phpr)$_3$)$_2$ MOP, $C_{80}H_{90}Cu_3O_{12}Si_2$ for n = 1 (*M* = 1490.37 g mol^{-1}):**<br>$\bar{V}_c$ = {(80 × 9.1) + (90 × 3.1) + (3 × 7.1) + (12 × 5.5) + (2 × 26.1)} + 12.4 − (12 × 8.1) − (3 × 14.1) = 1083.4 cm^3 mol^{-1}<br>Estimated density = 1.376 g cm^{-3}; estimated partial specific volume $\bar{v}$ = 0.727 cm^3 g^{-1}.

The experimental $\bar{v}$ value for the known Cu_4(*m*-pbhx)$_4$ molecular square is 0.886 cm^3 g^{-1}, compared with the D-Z estimated value of 0.839 cm^3 g^{-1}. Based on this result, the partial specific volumes for the Cu-Si compounds are likely to be slightly higher than those obtained from D-Z values. Approximate $\bar{v}$ values for the new Cu-Si MOPs can be obtained by adding 0.047 cm^3 g^{-1} to the D-Z values, or by multiplying the D-Z values by 0.886/0.839. The suggested values listed below are the averages of the two corrected results (which differed by less than 0.01 cm^3 g^{-1} in each case).

[Cu_3(MeSi(phac)$_3$)$_2$]$_n$: 0.716 cm^3 g^{-1}

[Cu_3(MeSi(phpr)$_3$)$_2$]$_n$: 0.771 cm^3 g^{-1}

These values are in the order [Cu_3(MeSi(phac)$_3$)$_2$]$_n$ < [Cu_3(MeSi(phpr)$_3$)$_2$]$_n$ < Cu_4(*m*-pbhx)$_4$. This order is reasonable because [Cu_3(MeSi(phac)$_3$)$_2$]$_n$ has the smallest fraction of H atoms of the three compounds, and thus is expected to have the highest density and the lowest $\bar{v}$.

2. Partial specific volumes for the different [Cu_3L_2]$_n$ oligomers

The D-Z method yields the same estimate of $\bar{v}$ for the different oligomers [Cu_3(CH_3Si(phac)$_3$)$_2$]$_n$, because they all have the same constituents in the same ratios. However, the experimental partial specific volumes of the oligomers may not all be the same. The smaller oligomers (n = 1-3) have smaller internal volumes and smaller pore sizes as compared to the larger molecules. This could make the smaller oligomers less accessible to solvent, which would increase their partial specific volumes.

For example, if the smallest oligomer, [Cu_3(CH_3Si(phac)$_3$)$_2$], has a larger partial specific volume than the D-Z estimate, the value of *M* calculated from AUC data will be too small. If the

correct value of $\bar{v}$ for this compound is 0.9 cm^3 g^{-1}, then the AUC data summarized in Table 1 would yield values of M_1 of 690 g mol^{-1} (in toluene) and 1800 g mol^{-1} (in fluorobenzene). Similarly, a value of $\bar{v}$ of 0.88 cm^3 g^{-1} for [$Cu_3(CH_3Si(phpr)_3)_2$] would yield values of M_1 closer to the calculated molecular weight.

Choosing larger $\bar{v}$ increases the calculated M values from the fluorobenzene experiments faster than those in toluene, because of the greater density of fluorobenzene. This exacerbates the differences between calculated M values in the two solvents. One could propose that $\bar{v}$ is not the same in the two solvents. However, this would run counter to our experiments with the Cu molecular square [Cu_4(*m*-pbhx)$_4$], which gave very similar values of $\bar{v}$ in four different solvents.

The combinations of solutes and solvents studied here have $\bar{v}\rho$ close to 1. This situation increases the sensitivity of the calculated M values to small changes in $\bar{v}$. This problem could be mitigated by using solvents of lower density for the AUC measurements. However, the MOPs are not soluble in common lower-density solvents (such as alkanes and ethers).

References

S1. Durchschlag, H.; Zipper, P., Calculation of Partial Specific Volumes and Other Volumetric Properties of Small Molecules and Polymers. *J. Appl. Crystallogr.* **1997,** *30*, 803–807.

S2. Durchschlag, H.; Zipper, P., Calculation of the Partial Volume of Organic Compounds and Polymers. *Prog. Colloid Polym. Sci.* **1994**, *94*, 20–39.
